# Forkhead Box S1 mediates epithelial-mesenchymal transition through the Wnt/β-catenin signaling pathway to regulate colorectal cancer progression

**DOI:** 10.1186/s12967-022-03525-1

**Published:** 2022-07-21

**Authors:** Liang Zhang, Chuan-fu Ren, Zhi Yang, Long-bo Gong, Chao Wang, Min Feng, Wen-xian Guan

**Affiliations:** 1 Department of General Surgery, Drum Tower Clinical Medical College of Nanjing Medical University, 321 Zhongshan Road, Nanjing, 210008 Jiangsu People’s Republic of China; 2 Department of Gastrointestinal, Xuzhou Central Hospital, Affiliated Central Hospital of Xuzhou Medical University, Xuzhou, Jiangsu China

**Keywords:** FOXS1, Wnt/β-catenin signaling pathway, Colorectal cancer, Epithelial–mesenchymal transition

## Abstract

**Background:**

Recent studies have shown that the fox family plays a vital role in tumorigenesis and progression. Forkhead Box S1 (FOXS1), as a newly identified subfamily of the FOX family, is overexpressed in certain types of malignant tumors and closely associated with patient's prognosis. However, the role and mechanism of the FOXS1 in colorectal cancer (CRC) remain unclear.

**Method:**

FOXS1 level in CRC tissues and cell lines was analyzed by western blot and quantitative real-time polymerase chain reaction (qRT-PCR). Immunohistochemistry (IHC) was used to detect the relationship between FOXS1 expression and clinicopathological features in 136 patients in our unit. The expression of FOXS1 was knocked down in CRC cells using small interfering RNA (siRNA) technology. Cell proliferation was assessed by CCK8 assay, colony formation, and 5-Ethynyl-20-deoxyuridine (EdU) incorporation assay. Flow cytometry detected apoptosis and wound healing, and Transwell assays determined cell migration and invasion. Western blotting was used to detect the levels of proteins associated with the Wnt/β-catenin signaling pathway. Then, we used short hairpin RNA (shRNA) to knock down FOXS1 to see the effect of FOXS1 on the proliferation, migration, invasion, and metastasis of CRC cells in vivo. Finally, we investigated the impact of Wnt activator LiCl on the proliferation, migration, invasion, and metastasis of CRC cells after FOXS1 knockdown.

**Result:**

Compared to those in normal groups, FOXS1 overexpressed in CRC tissues and CRC cells (P < 0.05). Upregulation of FOXS1 association with poor prognosis of CRC patients. si-FOXS1 induced apoptosis and inhibited proliferation, migration, invasion, the epithelial-mesenchymal transition (EMT), and the Wnt/β-catenin signaling pathway in vitro; sh-FOXS1 inhibited the volume and weight of subcutaneous xenografts and the number of lung metastases in vivo. LiCl, an activator of Wnt signaling, partially reversed the effect of FOXS1 overexpression on CRC cells.

**Conclusion:**

FOXS1 could function as an oncogene and promote CRC cell proliferation, migration, invasion and metastasis through the Wnt/βcatenin signaling pathway, FOXS1 may be a potential target for CRC treatment.

## Introduction

Colorectal cancer (CRC) is the third most common cancer and the fourth most common cause of oncological death worldwide [1]. As a public health problem, there are 1.4 million new cases of CRC each year, accounting for more than 9% of the total cancer incidence [2, 3]. In China, the incidence of CRC is also on the rise as the economy improves and the diet and lifestyle become more westernized. The development of CRC is a long-term chronic process. It undergoes a three-way transformation from inflammation to adenoma to cancer, and early detection and diagnosis is the key to improving long-term prognosis. CRC is currently considered a heterogeneous disease at the cellular and molecular levels, with environmental factors, epigenetic susceptibility, and specific molecular mechanisms involved in the development of CRC. Due to the high molecular heterogeneity of CRC, screening for additional diagnostic and prognostic biomarkers and therapeutic targets is critical to improving clinical outcomes.

Transcription Factors (TFs) are proteins that bind DNA in a sequence-specific pattern and regulate transcription. It is now known that dysregulation of TFs leads to altered gene expression, which promotes cell differentiation/proliferation, migration, and metastasis, as well as decreases chemotherapeutic drug sensitivity, and is a crucial determinant of tumor biological behavior [4]. The FOX gene family of TFs is an evolutionarily conserved group of transcriptional regulators consisting of 50 encoding genes, 19 subfamilies ranging from FOXA to FOXS based on the degree of homology of their forkhead structural domains. They regulate numerous biological functions in human development and adulthood [5]. Variants in the FOX TF family of genes have been shown to contribute to human disease. It has been shown that variations in some FOX gene subfamilies are associated with the development of multiple cancer types [6], further highlighting the importance of the FOX family TFs as molecular agents. Currently, 19 subfamilies of the FOX gene family are known, 14 of which are associated with the development of colon cancer. FOXS1, the most recent subfamily, is essential for forming the testicular vasculature [7] and is related to human hepatocellular and gastric cancers [8, 9]. Whereas the relationship between the FOXS1 gene and human tumors is still very poorly studied. The role of the FOXS1 gene in human CRC is even less clear.

CRC often takes more than 10 years to develop from normal colonic epithelium to a malignant phenotype, accompanied by many genetic changes related to the Wnt/β-catenin signaling pathway [10]. β-catenin is a critical regulatory protein in the Wnt/β-catenin signaling pathway, and approximately 1% of CRC patients will have β-catenin mutations. High expression of β-catenin in the body suggests poor prognosis. Matrix Metallopeptidase 7(MMP7), another downstream target protein of the Wnt/β-catenin signaling pathway, is found in over 90% of colorectal tissues and is associated with poor prognosis. In more than 94% of CRC cases, at least one Wnt/β-catenin signaling pathway protein is known to be mutated [11]. EMT has been considered a significant factor in tumor invasion and metastasis. In EMT, CRC cells Loss epithelial properties (e.g., protein E-cadherin expression, β-Catenin is down-regulated in the nucleus) and gain mesenchymal properties (e.g., vimentin, fibronectin, etc. are upregulated) with a decrease in cell adhesion, a change in shape to a spindle-shaped fibrous cell morphology to accommodate the motility requirements [12]. Current literature suggests that Wnt/β-catenin pathway is essential for EMT of malignant tumors [13]. Activation of the WNT pathway contributes to phenotypic changes in tumor cells and promotes apoptosis resistance, invasion, and metastasis. However, whether the FOXS1 contributes to CRC progression and metastasis through a mechanism involving the WNT pathway and EMT remains unclear.

Therefore, this study aimed to investigate the relationship between the FOXS1 gene and the clinical characteristics and prognosis of CRC and investigate the molecular mechanisms involved.

## Materials and methods

### Patient samples

Eight pairs of CRC tissues and adjacent tissues were obtained from Nanjing Drum Tower Hospital (Nanjing, China) from March 2021 to April 2021. Total proteins were immediately extracted from these clinical tissues. For immunohistochemical (IHC) analysis of FOXS1 protein expression, we randomly selected 136 formalin-fixed paraffin-embedded CRC patients' tissues and their corresponding follow-up data from January 2014 to December 2014 in Nanjing Drum Tower Hospital. All patients provided written informed consent. This study was performed following the principles of the Declaration of Helsinki. The Ethics Committee of Nanjing Drum Tower Hospital approved this study.

### Bioinformatic analysis

The latest transcriptome sequencing and clinical data of colon cancer were downloaded from The Cancer Genome Atlas (TCGA; https://portal.gdc.cancer.gov/), including 471 cancer samples and 41 paracancerous samples. The R package survminer 0.4.8 was used to calculate the best cut off value of FOXS1, which classified colon cancer tissues into FOXS1_low and FOXS1_high groups, and plot the survival curves. The R package limma 3.44.3 was utilized to perform differential analysis between the two groups. Then all genes were sorted from high to low according to the difference fold. The R package clusterProfiler 3.16.1 was used to perform gene set enrichment analysis (GSEA). All the gene sets were download from mSigDB (https://www.gsea-msigdb.org/gsea/index.jsp).

### Cell line and culture

The human immortalized colonic epithelial cell line (NCM460) and human CRC lines (LoVo, SW620, DLD1, RKO) were purchased from Shanghai Cafa Biological Technology Co., Ltd. (Shanghai, China). Cells were cultured in Dulbecco’s modified Eagle’s medium (DMEM, Gibco, Waltham, MA, USA) with 10% fetal bovine serum (Gibco, Waltham, MA, USA) at 37 °C, 5% CO2 condition.

### Cell transfection with small interfering RNA (siRNA)

To transiently knockdown the expression of FOXS1, FOXS1-siRNAs (siRNA#1, 5′-CAGGAAUGUUCUUUGATT-3′, 5′-UCAAAGAAGAACAUUCCUGTT-3′; siRNA#2, 5′-CACUCAACGAGUGCUUUGUTT-3′, 5′-ACAAAGCACUCGUUGAGUGTT-3′; siRNA#3, 5′-GGCCAAUAAAGCCAUGUGATT-3′, 5′-UCACAUGGCUUUAUUGGCCTT-3′) or control-siRNA were transfected into SW620 and LoVo cells. Shanghai GenePharma Company (Shanghai, China) provided these siRNAs. 2 × 10^5^ CRC cells per well plated into a six-well plate were treated with siRNA (1–2 µg) encapsulated by the interferin reagent (Polyplus, New York, NY, USA) based on the protocol. The knockdown efficiency of FOXS1 siRNA was examined by western blot. FOXS1 shRNA (Shanghai Gene Chem Co, Ltd., China) was used to establish stable cell lines to knock down the expression of FOXS1.

### Quantitative reverse transcription polymerase chain reaction (qRT-PCR)

Total RNA from CRC cell lines (LoVo, SW620, DLD1, RKO) and human normal colon NCM460 cells was extracted by Trizol Reagent (1596-026, Invitrogen) and reverse-transcribed into cDNA using an RT-PCR kit (Takara, Kyoto, Japan). The sequences of the primers were as follows: FOXS1-forward, 5ʹ- AGTGGCATCTACCGCTACATC-3 and FOXS1-reverse, 5ʹ- CACCTTGACAAAGCACTCGT-3ʹ; human GAPDH Forward: 5’- GGAGTCCACTGGCGTCTTCA-3′ and Reverse.: 5’- GGGGTGCTAAGCAGTTGGTG-3′. The RT-PCR assays were performed using SYBR Green Premix Ex Taq on an ABI ViiA 7DX RT-PCR machine. Human GAPDH was used as an internal reference gene. Relative mRNA expression was calculated according to the 2-(△Ct△Ct) method.

### Western blot analysis

Total proteins were extracted from CRC tissue samples or cells with RIPA lysis buffer (Beyotime Institute of Biotechnology, Jiangsu, China) containing protease inhibitors. The bicinchoninic acid (BCA) protein assay kit (Pierce, Rockford, USA) was used to measure protein concentrations. Antibody information is as follows: FOXS1 (ThermoFisher, PA5-49703), GAPDH (Abcam, ab9485), β-tubulin(Cell Signaling Technology, #2146S), E-Cadherin (Cell Signaling Technology, #3195S), N-Cadherin (Cell Signaling Technology, #13116S), Vimentin (Cell Signaling Technology, #5741S), MMP9 (ProteinTech Group, #10375-2-AP), β-catenin(ProteinTech Group, 51067-2-AP), GSK-3β(ProteinTech Group, 22104-1-AP), TCF7(ProteinTech Group, 14464-1-AP), MMP7(ProteinTech Group, 10374-2-AP), C-Myc(ProteinTech Group, 10828-1-AP) The bands of interest in the Western blots were normalized to GAPDH or tubulin.

### IHC staining and scoring

The expression pattern of FOXS1 in tumorous colorectal tissues was assessed using IHC method [14]. Two experienced pathologists used a double-blind method to assess FOXS1 expression under five randomly selected high magnification stains. Staining results were quantified according to staining intensity and percentage of FOXS1-positive cells. IHC staining results were graded according to the following criteria [15]: staining intensity score: 0 (negative), 1 (weak expression), 2 (moderate expression) and 3 (strong expression); while the proportion of FOXS1-positive cells staining area was scored as follows: 0 (0%), 1 (1–25%), 2 (26–50%), 3 (51–75%) and 4 (76–100%). The final staining score was calculated as the proportion of positive tissue multiplied by the staining intensity score (range 0 ~ 12). A staining index score of ≥ 6 was considered high FOXS1 expression, and ≤ 4 was considered low FOXS1 expression.

### Cell counting kit-8 (CCK-8) assay

Cell proliferation measured by CCK-8. LoVo and SW620 cells were plated in 96-well plates at a density of 2 × 10^3^/well (90 μl/well), incubated for 24 h, 48 h, and 72 h then 10 μl of CCK8 reagent was added to each well. The cells were incubated with 10% CCK-8 at 37 °C for 2 h, then the absorbance was detected at 450 nm.

### Colony formation assay

The logarithmic growth cells were seeded into 6-well plates (1000 cells/well) and cultured at 37 °C with 5% CO2 for 10–14 days. The colonies were fixed in methanol for 30 min and stained with 1% crystal violet (Beyotime, Nanjing, China). Cell colonies containing more than 50 cells were counted.

### 5-Ethynyl-20-deoxyuridine (EdU) incorporation assay

According to the manufacturer's protocol, the EdU assay was performed with a Cell-Light EdU DNA Cell Proliferation Kit (C0071L, Beyotime, Shanghai, P.R. China). After incubation with 10 µM Edu for 2 h, the CRC cells were fixed with methanol for 15 min at 37 °C and then washed with Phosphate Buffered Saline (PBS) containing 3% BSA for 3 × 5 min. The fixed cells were soaked in permeabilizing solution (PBS containing 0.3% Triton X-100) for 10–15 min and then washed 3 times with PBS containing 3% Bovine Serum Albumin (BSA) extensively. Next, the cells were incubated with Click Additive Solution for 30 min in the dark and washed with PBS containing 3% BSA for 3 × 5 min. The cells were incubated with Hoechst 33342 for 10 min in the dark to stain the nuclei. Finally, the EdU-positive cells were photographed and counted under an Olympus FSX100 microscope (Olympus, Tokyo, Japan) in five randomly selected fields.

### Wound healing assay

5 × 105 Cells were inoculated in 6-well plates and cultured to 90% confluency, scratches were performed into the middle slides using a sterile 200ul pipette tip and then cultured with fresh Dulbecco's Modified Eagle Medium (DMEM) medium without serum for 48 h. The scratch zones were determined via a microscope at 0 and 48 h. Cell migration was calculated as the percentage of wound closure.

### Transwell migration and invasion assays

The transwell apparatus (Corning, NY, USA) with the upper chamber of an 8-μm pore size polycarbonate membrane was used to perform cell transwell migration and invasion assays. For migration assays, CRC cells (1 × 10^5^ cells) were resuspended in a 200 μL (Fetal Bovine Serum) FBS-free medium and added to the upper chamber; 600ul DMEM containing 10% fetal bovine serum was added into the lower chamber. After incubation at 37 °C for 24 h, we fixed and stained the cells and counted the number of cells in six randomly selected fields under the microscope. For invasion assays, cells (2 × 105) in a 200 μL FBS-free medium were seeded into the upper chamber with a thin layer of 0.5 mg/L Matrigel, 600ul DMEM containing 10% fetal bovine serum was added into the lower chamber.

### Apoptosis assay

For flow-through detectionAnnexin-V-AbFlour™ 647 Apoptosis Detection Kit solution (Abbkine, Wuhan, Hubei, China) was used to detect the cell apoptotic rate. A total of 1 × 10^5^ resuspended cells were taken, centrifuged at 1000 g for 5 min, the supernatant was discarded, and 195 μl Annexin V-FITC conjugate was added to gently resuspend the cells, followed by 5 μl Annexin V-FITC and 10 μl propidium iodide staining solution and gently mixed. Incubate for 10–20 min at room temperature (20–25 °C) protected from light, followed by flow-through assay.

### TUNEL reaction

Cell apoptosis in mouse colorectal tissue was determined by terminal deoxynucleotidyl transferase (TdT)-mediated dUTP nick and labeling (TUNEL) staining according to the manufacturer's instructions. TUNEL-positive cells were detected using fluorescence microscopy.

### Tumour xenograft and metastasis in vivo

Subcutaneous tumor formation model: 12 nude mice at 4–6 weeks were randomly divided into control group and knockdown group, the control group was injected with Sh-NC transfected cells subcutaneously, and the knockdown group was injected with Sh-FOXS1 transfected cells subcutaneously. After 21 days of growth, nude mice were sacrificed by cervical dislocation, subcutaneous tumors were removed, weights were measured, and tumor sizes (calculated volume = shortest diameter^2^ *longest diameter/2) were measured every 5 days during the observation period. Lung metastasis model in nude mice: Sh-NC transfected cells and Sh-FOXS1 transfected cells were injected intravenously via the tail margin, observed to grow for 21 days, nude mice were sacrificed by cervical dislocation, lungs were removed, and the number of metastatic nodules in the lungs was counted.

### Statistical analysis

GraphPad Prism (version 6; La Jolla, CA, USA) and SPSS statistics software (version 23; IBM, Armonk, NY, USA) were used for statistical analysis. Continuous data were presented as means ± standard deviation (SD), Student’s *t*-test, one-way ANOVA, two-way ANOVA, and Pearson correlation analysis were used in this study. Kaplan-Meier analysis and the log-rank test were used for survival analysis. *p* < 0.05 was considered statistically significant.

## Results

### High FOXS1 expression was significantly associated with poor prognosis.

**Figure1:**
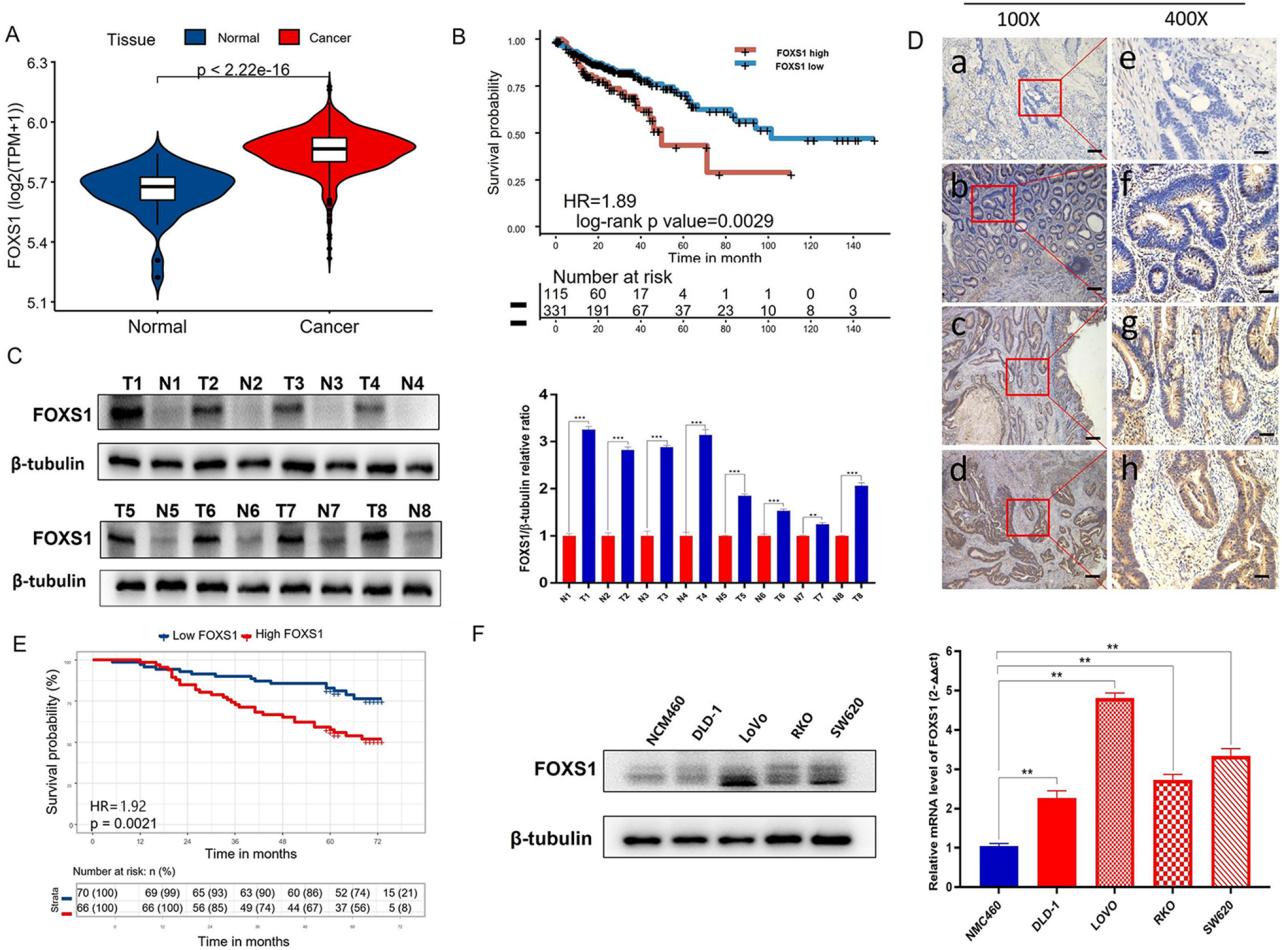
FOXS1 is overexpressed in primary colorectal cancer (CRC). **A** The FOXS1 mRNA was significantly upregulated in CRC compared with adjacent normal tissues (P < 0.01). **B** Kaplan–Meier analysis indicated that CRC patients with high FOXS1 expression had significantly worse OS than patients with low FOXS1 expression (P < 0.01). **C** The protein levels of FOXS1 in 8 pairs of colorectal cancer and paracancer tissues. **D** The expression of FOXS1 protein was distributed in the cytoplasm and nucleus of CRC tumor tissues; a and e, FOXS1 was not detected in normal colorectal tissues; b and f, representative images of weak FOXS1 staining in CRC tissues; c and g, representative images of moderate FOXS1 staining in CRC tissues; d and h, representative images of strong FOXS1 staining in CRC tissues; a, b, c, and d, original magnification ×100 (scale bar = 100 μM); e, f, g, and h, ×400. **E** The higher expression of FOXS1 was related to shorter OS in CRC patients. **F** Western blot and qPCR analysis of FOXS1 expression in various CRC cell lines and normal colon epithelial cell line NCM460

To explore the potential value of FOXS1 in the therapeutic target of CRC, we analyzed FOXS1 expression in the TCGA cohort; the results suggested that FOXS1 was significantly upregulated in CRC compared with adjacent normal tissues (P < 0.01, Fig. 1A). Moreover, Kaplan-Meier analysis indicated that CRC patients with high FOXS1 expression had significantly worse overall survival (OS) than patients with low FOXS1 expression (P < 0.01, Fig. 1B). To validate the results of TCGA data analysis, we detected the protein levels of FOXS1 in 8 pairs of CRC and paracancer tissues. As shown in Fig. 1C, FOXS1 was highly expressed in CRC tissues at both protein levels in comparison to paracancerous tissues. To assess the relation between FOXS1 expression and clinicopathological features, we performed IHC staining on 136 cases of paraffin-embedded CRC tissues. The expression of FOXS1 protein was distributed in the cytoplasm and nucleus of CRC tumor cells but was negative in normal colonic tissue cells (Fig. 1D). The higher expression of FOXS1 was related to shorter OS in CRC patients (Fig. 1E). In addition, we also found that upregulated FOXS1 expression was correlated with Differentiation degree (P = 0.01), N stage (P = 0.029) and TNM stage (P < 0.001) (Table 1). Furthermore, we assessed mRNA and protein levels of FOXS1 in four CRC cell lines (DLD1, SW620, LoVo, RKO) and one human normal colon NCM460 cell. Our data show that FOXS1 is overexpressed in CRC cell lines compared with NCM460 cells (Fig. 1F). The above results show that FOXS1 is highly expressed in CRC and is associated with the long-term prognosis of CRC, which could be a potential therapeutic target in CRC.

**Table 1:** Clinicopathological characteristics of 136 Patients with CRC

Characteristics	Number	Low(Foxs1)	High(Foxs1)	p valuve
Gender				0.56
Male	78	38	40	
Female	58	32	26	
Age				0.36
< 60 years	56	32	24	
> 60 years	80	38	42	
Differentiation degree				0.01*
Well	4	2	2	
Morderate	108	62	46	
Poor	24	6	18	
Tumor location				0.503
Ascending colon	30	17	13	
Tranverse colon	8	5	3	
Descending colon	6	3	3	
Sigmoid colon	36	14	22	
Rectum	56	31	25	
T stage				0.079
1	4	4	0	
2	18	12	6	
3	103	50	53	
4	11	4	7	
N stage				0.029*
0	69	43	26	
1	49	21	28	
2	18	6	12	
M stage				0.108
0	130	69	61	
1	6	1	5	
TNM stage				< 0.001*
I	15	11	4	
II	53	32	21	
II	62	30	32	
IV	6	1	5	

^*^p < 0.05 were considered statistically signifcant.

### Knockdown of FOXS1 inhibits the biological behavior, apoptosis resistance, and EMT in CRC

**Fig. 2:**
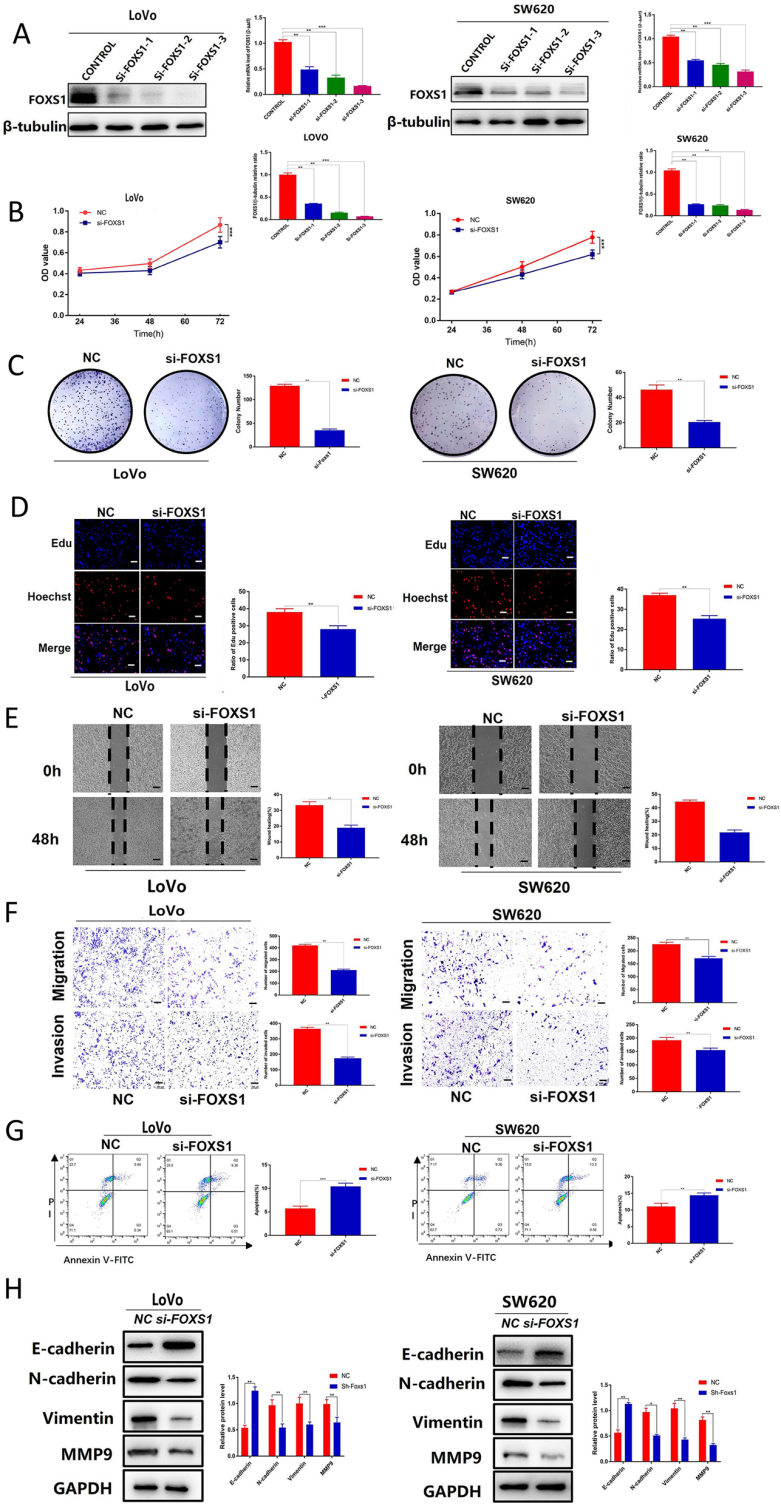
Knockdown of FOXS1 inhibits the biological behavior, apoptosis resistance, and EMT in CRC. **A** PCR and western blot were used to evaluate the knockout efficiency of the FOXS1-specific small interfering RNAs (siRNAs) (siFOXS1-1, siFOXS1-2, and siFOXS1-3) in LoVo and SW620 cells. **B** CCK-8, Colon formation (**C**) and (**D**) Edu assays were used to evaluate the proliferation of LoVo and SW620 cells after transfection with siFOXS1 for 48 h. **E** Cell mobility analyzed using a wound healing assay. **F** Migration and invasion of siFOXS1#3 and Control cells were detected using Transwell migration and invasion assays. **G** Propidium iodide (PI) and Annexin V staining levels were determined by flow cytometry to assess cell apoptosis in LoVo and SW620 cells transfected with siFOXS1 and Control. **H** Western blot analysis of E-cadherin, N-cadherin, vimentin, MMP9 protein expression in LoVo and SW620 cells transfected with FOXS1-siRNA. Results were shown as mean ± SD of three independent experiments, each experiment was performed in triplicate. *P < 0.05; **P < 0.01

LoVo and SW620 were selected for the following experiments because of the high mRNA and protein expression levels of FOXS1. To further explore the biological functions of FOXS1in CRC, we knocked down LoVo and SW620 cells with control siRNA and FOXS1-siRNAs (si#1, si#2, and si#3). The protein levels of FOXS1 were significantly downregulated after knockdown, with si-FOXS1#3 having a higher knockdown efficiency than si-FOXS1#1–2 (Fig. 2A), so si-FOXS1#3 was used for subsequent studies. Knockdown of FOXS1 significantly inhibited cell proliferation (Fig. 2B–D), migration (Fig. 2E, F), invasion (Fig. 2F) and apoptosis resistance (Fig. 2G) of both LoVo and SW620 cells. In addition, we also find that FOXS1 knockdown induced an increase in E-cadherin levels with a concomitant decrease in N-cadherin and Vimentin (Fig. 2H). Together, our above data suggest that FOXS1 inhibited the biological behavior, apoptosis resistance, and EMT in CRC.

### FOXS1 Is a Regulator of the Wnt/β-catenin Pathway

**Fig. 3:**
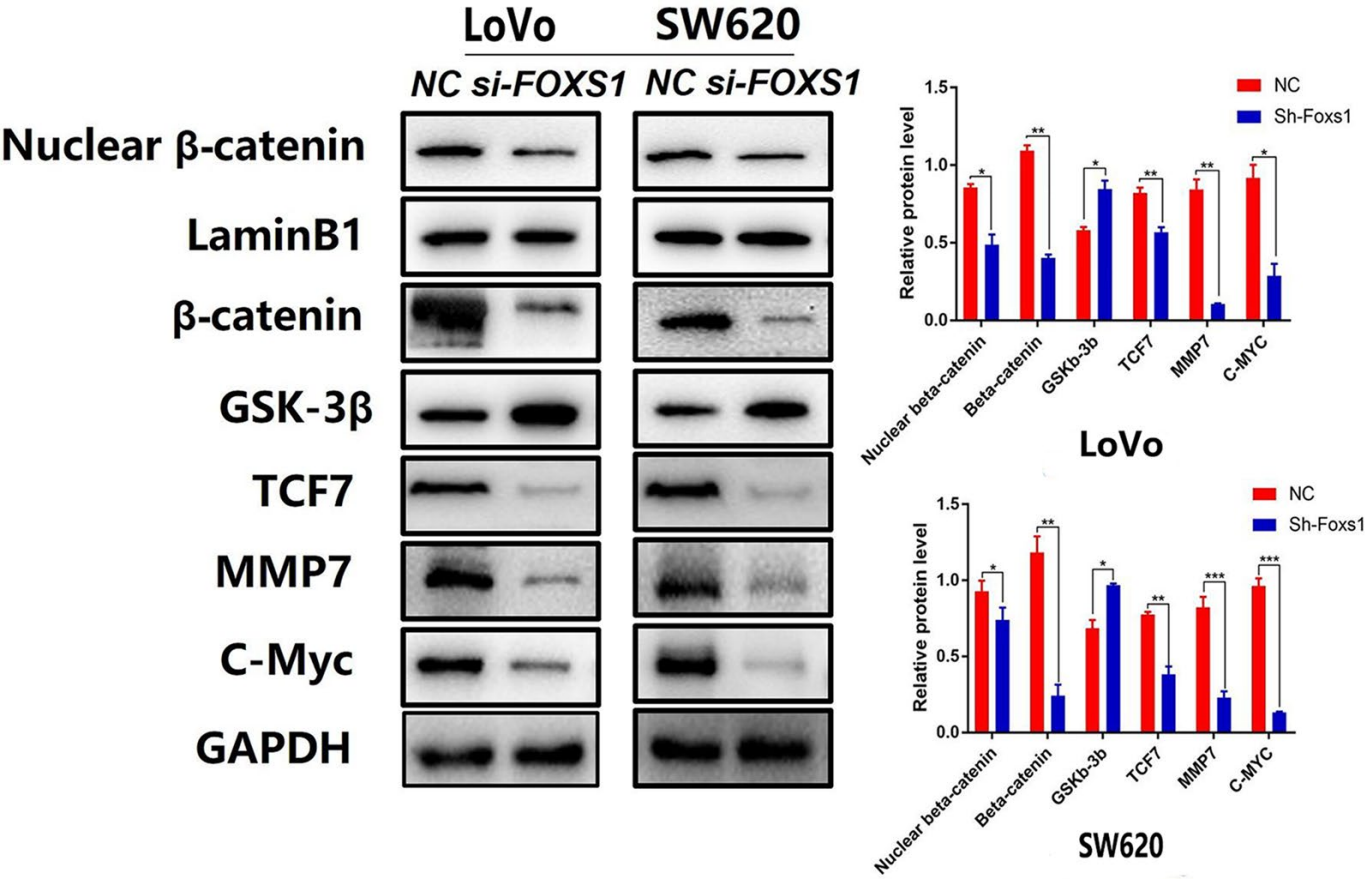
FOXS1 is a regulator of the Wnt/β-catenin Pathway. Western blot analysis of Nuclear β-catenin, β-catenin, GSK-3β, TCF7, MMP7, C-Myc protein expression in LoVo and SW620 cells transfected with FOXS1-siRNA. Results were shown as mean ± SD of three independent experiments, each experiment was performed in triplicate. *P < 0.05; **P < 0.01

We also investigated the underlying molecular mechanism of FOXS1 in EMT. Current literature suggests that Wnt/β-catenin pathway is essential for EMT of gastrointestinal cancer [10, 11] and half of all mammalian FOX transcription factors have been assigned a role in the Wnt pathway [16], primarily in cancer cells. Given these considerations, we analyzed Wnt/β-catenin signaling pathway-related proteins in LoVo and SW620 cells 48 h after transfection with or without siRNA by Western blotting. The result showed that FOXS1 knockdown reduced the levels of total β-catenin and the nuclear translocation of β-catenin as well as the Wnt/β-catenin target genes (c-Myc, TCF7, MMP7) in FOXS1-deficient cells (Fig. 3). These data indicated that FOXS1 knockdown downregulated β-catenin and β-catenin target genes (c-Myc, TCF7, MMP7) expression, and FOXS1 may be involved in CRC carcinogenesis by activating the Wnt/β-catenin signal pathway.

### Effects of FOXS1 in CRC depend on Wnt/β-catenin signaling

**Fig. 4:**
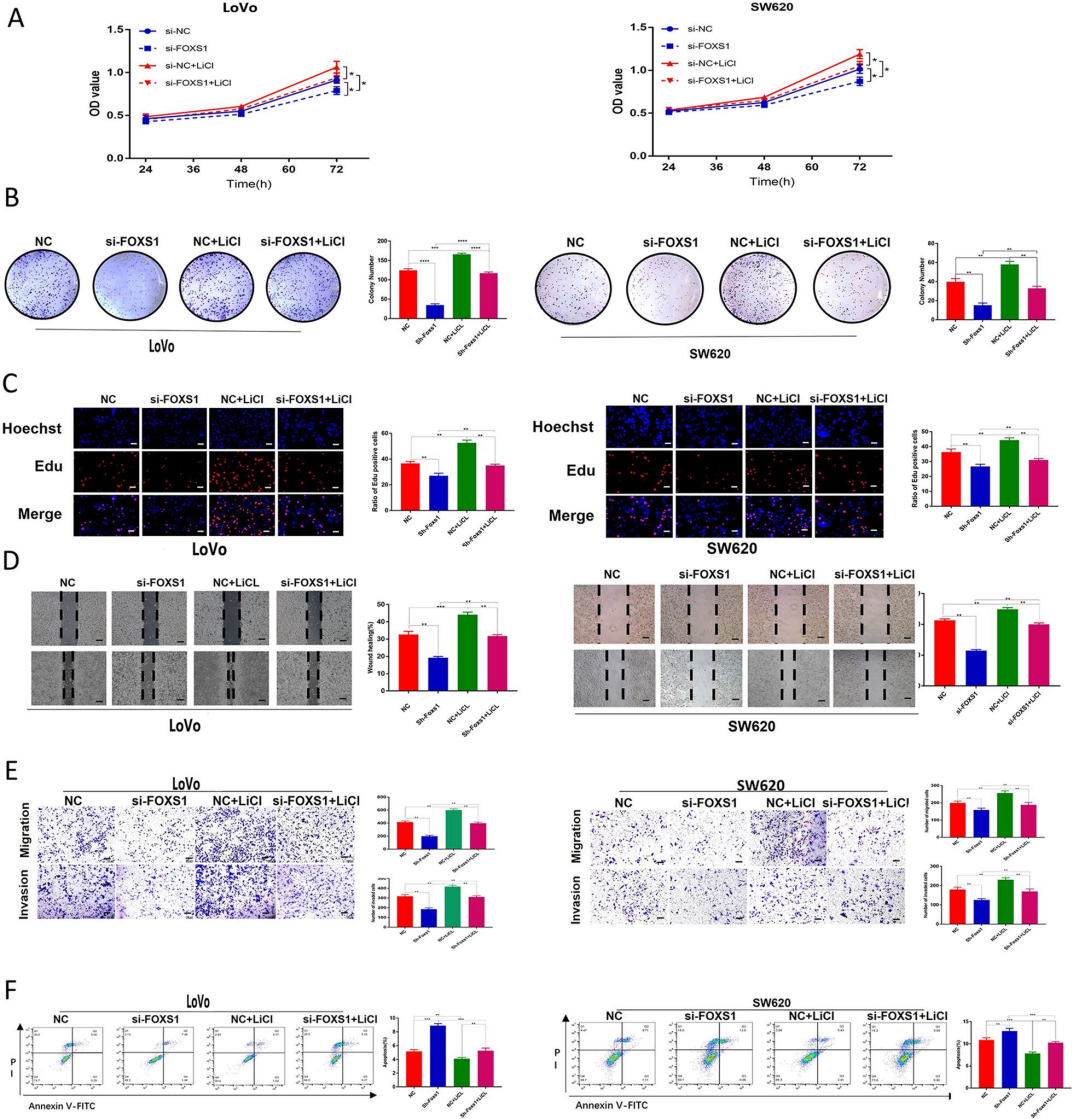
Inhibition of FOXS1 suppresses cell migration, invasion, proliferation, and increases apoptosis in CRC cells. **A** CCK8 **B**, colony formation **C**, Edu assays **D**, Wound healing **E**, transwell assays were performed to detect the migration, invasion, and proliferation ability of CRC cells transfected with or without FOXS1 inhibition, in CRC cells, as well as with or without the Wnt pathway activator by LiCl. **F** Flow cytometry was used to assess cellular apoptosis of CRC cells transfected with or without si-FOXS1, as well as those of cells treated with or without LiCl. Data represent mean ± SD of three independent experiments. Data are shown as mean ± SD; ns, no significant difference; *p < 0.05, **p < 0.01, ***p < 0.001, based on Student’s *t*-test

To further validate the biological behavior of FOXS1 in CRC and the interaction with the Wnt/β-catenin pathway, we used LiCl (a GSK-3β inhibitor that activates the β-catenin-mediated Wnt signaling pathway) to reverse the inhibitory effect of FOXS1 knockdown on EMT and cell phenotype. CCK-8 assay, Edu thymidine analog incorporation assay, and colony formation were used to evaluate the proliferation of LoVo and SW620 cells. The proliferative capacity ability of CRC cells was significantly reduced in the shFOXS1 group and increased in the LiCl group, while LiCl restored FOXS1 knockdown' effects (Fig. 4A–C). Compared to the control group, wound healing, migration, invasion ability of CRC cells were significantly decreased in the shFOXS1 group and elevated in the LiCl group; LiCl reverses the effect of a decrease in migration and invasion capacity induced by FOXS1 knockdown (Fig. 4D, E). In addition, FOXS1 knockdown significantly increased apoptosis in CRC cells. However, LiCl significantly reduced apoptosis caused by FOXS1 knockdown (Fig. 4F).

**Fig. 5:**
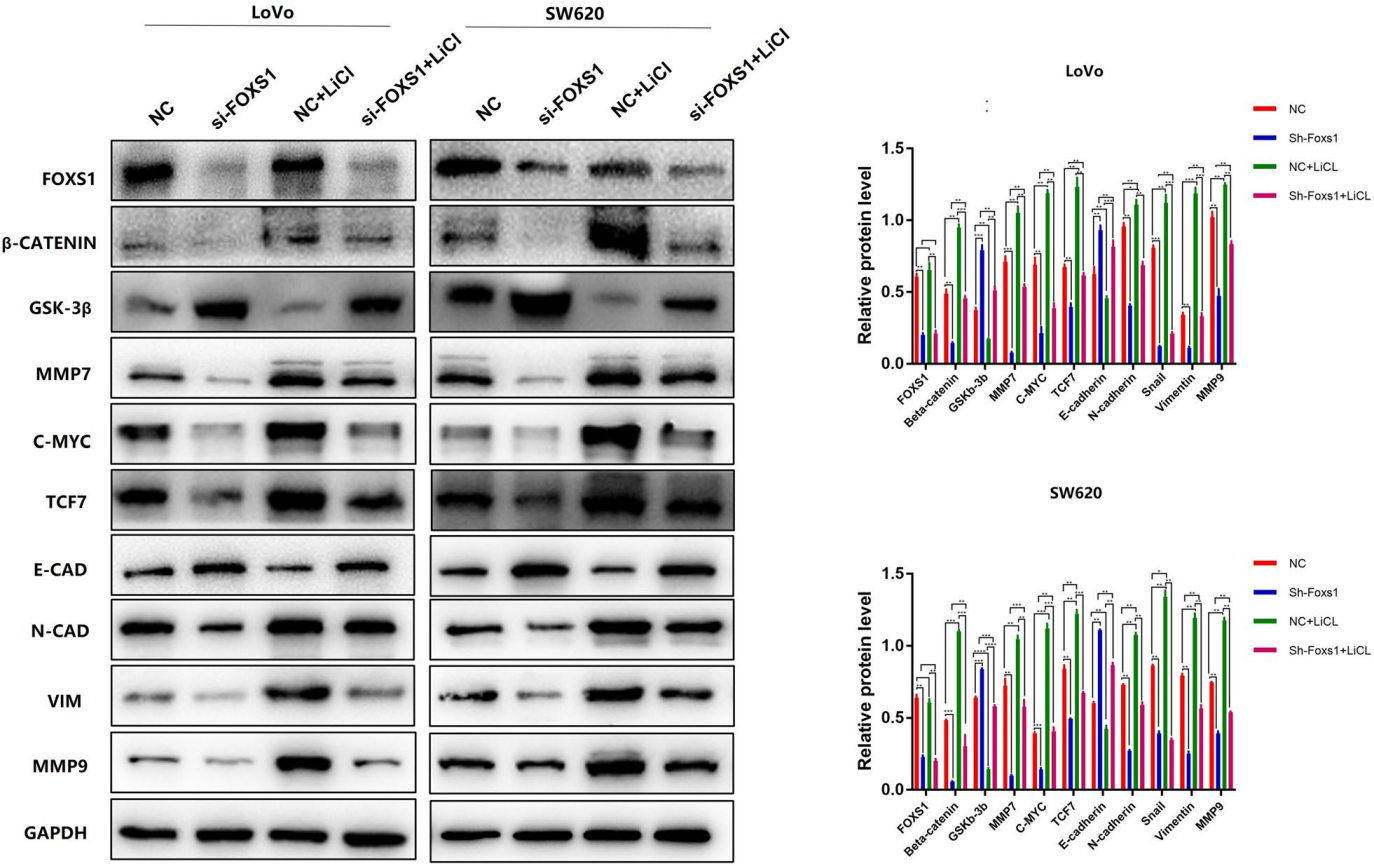
FOXS1 mediates EMT via Wnt/β-catenin signaling pathway in CRC. (**A**, **B** Western blot analysis the protein levels of FOXS1, Nuclear β-catenin, β-catenin, GSK-3β, TCF7, MMP7, C-Myc, E-cadherin, N-cadherin, vimentin, MMP9 in CRC cells transfected with or without si-FOXS1, as well as cells treated with or without the Wnt pathway activator LiCl

Subsequently, we observed a significant decrease of β-catenin and β-catenin target genes (c-Myc, TCF7, MMP7) and a significant increase of Gsk-3β in the FOXS1 knockdown group. In addition, FOXS1 knockdown increased E-cadherin levels while suppressing N-cadherin, MMP9, and Vimentin. This change was reversed by treatment with LiCl (Fig. 5A, B). These data demonstrate the essential role of FOXS1 in promoting EMT of CRC cells by activating the Wnt/β-catenin signaling pathway.

### FOXS1 promotes tumorigenicity and tumor metastasis in vivo

**Fig. 6:**
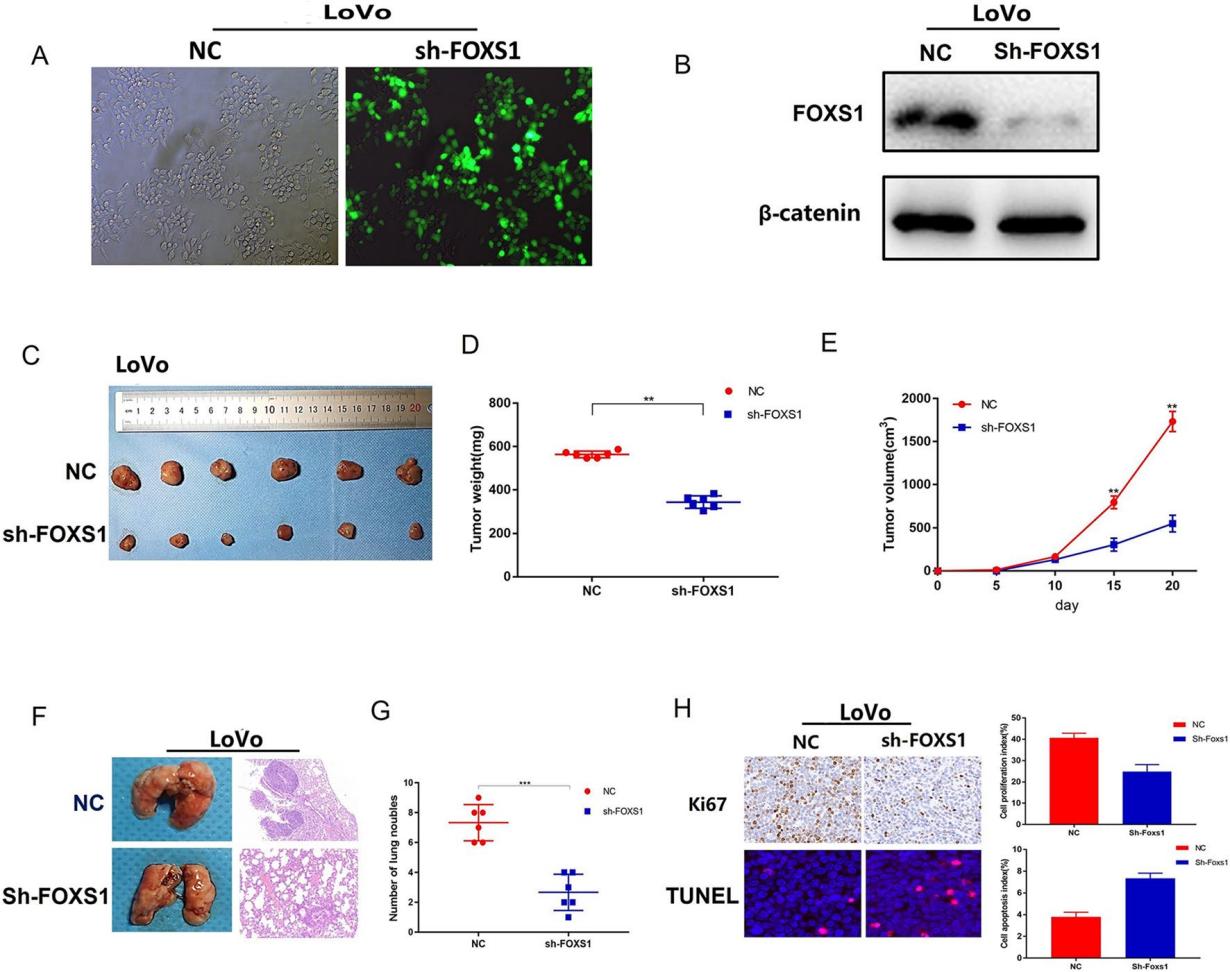
FOXS1 knockdown inhibited tumor growth and tumour metastasis in vivo. **A** Fluorescence was used to verify the infection efficiency (magnification × 200); **B** Western blot analysis of knockdown efficiency of shFOXS1; (**C–E**) Xenograft weight (mg) and size (cm) were measured; (**F**, **G**) Abolished tumour formation was found in the lungs of LoVo cell-injected mice. The statistical data of the tumour foci number are presented, H&E stained histological images of the lungs of the two groups of mice. **H** Ki67 and TUNEL were measured in xenograft tissues. *P < 0.05

To assess the effect of FOXS1 on tumor growth and metastasis in vivo, we constructed stably transfected LoVo cells (Fig. 6A, B) for subcutaneous xenografting in nude mice. After 21 days of growth, nude mice were sacrificed by cervical dislocation, the weight and volume were measured. As shown in Fig. 6C–E, FOXS1 knockdown markedly suppressed tumor growth in vivo compared to that of the controls. Then we examined the expression of Ki67 and Tunel in the xenografts by IHC. The results showed a significant decrease in Ki67 expression and a significant increase of apoptotic cells in the xenograft model derived from FOXS1 knockdown cells (Fig. 6H). Furthermore, we used a mice tail vein injection model to examine the effect of FOXS1 on tumor metastasis. The metastatic nodules in the lungs were determined 6 weeks after inoculation by hematoxylin and eosin (H&E) staining. The results revealed that FOXS1 knockdown suppressed the size and number of pulmonary metastatic nodules compared to the control group (Fig. 6F, G). Together, these data indicated that FOXS1 is involved in tumorigenesis and tumor metastasis in vivo.

## Discussion

Although various molecular markers have been reported to be involved in the diagnosis, treatment and prognosis of colorectal cancer patients [17, 18], there are still many molecular proteins that remain unexplored and need further research. The forkhead box (FOX) transcription factor family consists of a set of evolutionarily conserved transcriptional regulators involved in numerous functions during development and adulthood. Their dysfunction has been implicated in human diseases [19, 20]. It has been shown that variations in some FOX gene subfamilies are associated with the development of multiple cancer types [21, 22], further highlighting the importance of the FOX family TFs as molecular agents. At least 14 FOX subgroups have been associated with the pathogenesis of CRC, such as the FOXO subfamily, and a dual but paradoxical role for FOXO in CRC has been described, as they can act as cancer suppressor genes or oncogenes [23]. Expression levels of FOXM1 have been reported to correlate with cancer progression, lymph node and liver metastases, and high TNM staging. These findings are consistent with decreased patient survival [24]. FOXP3 is predominantly expressed by Treg cells, but it has also been found in other cells (e.g., Treg cells) and various normal tissues (e.g., breast, prostate, lung, thymus, colon, kidney, ovary). Due to the close relationship between Treg cells and the tumor microenvironment, FOXP3 has been extensively studied in the development and progression of CRC [25]. FOXS1, the most recent subfamily, is essential for the formation of the testicular vasculature [7] and associated with the prognosis of human hepatocellular and gastric cancers [8, 9]. However, the decisive contribution of FOXS1 to the invasion and migration of human CRC remains unclear. Our study is the first to show that FOXS1 was highly expressed in CRC and that FOXS1 was associated with the long-term prognosis of patients (Fig. 1). In addition, we revealed that FOXS1 promoted proliferation, migration, and invasion of CRC cells (Fig. 2). Then, We confirmed that FOXS1 is involved in tumorigenesis and tumor metastasis in vivo (Fig. 6).

Epithelial-mesenchymal transition (EMT) is a process in which epithelial cells acquire mesenchymal features and is often defined by the loss of the epithelial marker E-cadherin and the gain of the expression of the mesenchymal marker vimentin. The EMT program is widely recognized as a core component of carcinoma progression in cancer. EMT is also closely related to the invasion and metastasis of CRC. Our study demonstrated that FOXS1 knockdown could ameliorate the EMT process and malignant behavior of CRC cells. These results are consistent with the report that FOXS1 expression was inversely correlated with the expression of epithelial markers (E-cadherin) and positively correlated with the expression of mesenchymal markers (Vimentin and N-cadherin) in gastric and hepatocellular Carcinoma cells [8, 9].

Wnt/β-catenin signaling is a critical pathway regulating EMT in the development and progression of many kinds of malignancies [26–28]. In more than 94% of CRC cases, at least one Wnt/β-catenin signaling pathway protein is known to be mutated [11]. Thus, to evaluate the relationship between FOXS1 and Wnt/β-catenin signaling, we firstly investigated the levels of total β-catenin and the nuclear translocation of β-catenin as well as the Wnt/β-catenin target genes (c-Myc, TCF7, MMP7). The results showed that knockdown of FOXS1 attenuated Wnt/β-catenin related protein expression (Fig. 3).

**Fig. 7:**
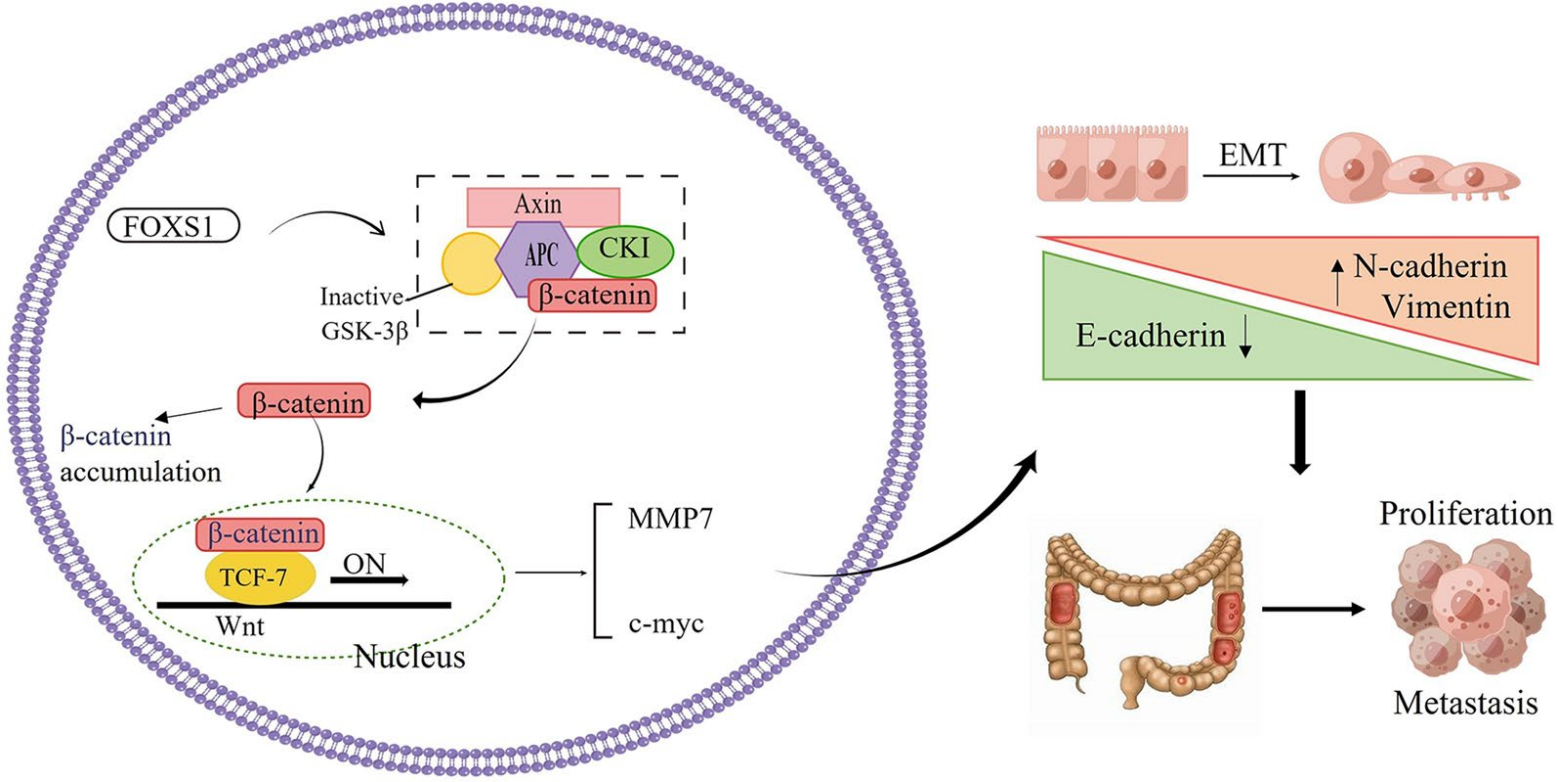
Schematic diagram illustrating the role of FOXS1 in promoting CRC proliferation, migration, invasion, and metastasis through the Wnt/β-catenin signaling pathway

Further, to demonstrate that Wnt/β-catenin acted as a critical pathway linking FOXS1 and EMT, we used LiCl (a GSK-3β inhibitor that activates the β-catenin-mediated Wnt signaling pathway) to upregulate the expression of β-catenin in FOXS1-deficient cells. Western blot analysis confirmed the β-catenin overexpression could neutralize the effects of FOXS1 decrease on EMT and reverse the changed EMT molecular markers. Collectively, these findings demonstrate the essential role of FOXS1 in promoting EMT by activating the Wnt/β-catenin signaling pathway in the development of CRC (Fig. 7).

## Conclusions

In summary, We first identified FOXS1 as an oncogene that promotes CRC proliferation, migration, invasion, and metastasis through the Wnt/β-catenin signaling pathway; the findings of this study may have important implications for the screening of meaningful predictive markers for CRC, and FOXS1 may be a new therapeutic target for CRC patients.

## Data Availability

All data generated or analysed during this study are included in this published article.

## Abbreviations

FOXS1: Forkhead Box S1
EdU: 5-Ethynyl-20-deoxyuridine
TFs: Transcription factors
GSEA: Gene set enrichment analysis
EMT: Epithelial-mesenchymal transition
qRT-PCR: Quantitative reverse transcription polymerase chain reaction
CRC: Colorectal cancer
IHC: Immunohistochemistry
FBS: Fetal bovine serum
TCGA: The Cancer Genome Atlas database
BSA: Bovine Serum Albumin
DMEM: Dulbecco's Modified Eagle Medium
PBS: Phosphate Buffered Saline
